# Spatial Impairment and Memory in Genetic Disorders: Insights from Mouse Models

**DOI:** 10.3390/brainsci7020017

**Published:** 2017-02-09

**Authors:** Sang Ah Lee, Valter Tucci, Giorgio Vallortigara

**Affiliations:** 1 Center for Mind/Brain Sciences, University of Trento, Rovereto 38068, Italy; giorgio.vallortigara@unitn.it; 2 Neuroscience and Brain Technologies Department, Istituto Italiano di Tecnologia, Genova 16163, Italy; valter.tucci@iit.it

**Keywords:** navigation, spatial memory, boundary geometry, feature, Prader-Willi, Beta-catenin gene

## Abstract

Research across the cognitive and brain sciences has begun to elucidate some of the processes that guide navigation and spatial memory. Boundary geometry and featural landmarks are two distinct classes of environmental cues that have dissociable neural correlates in spatial representation and follow different patterns of learning. Consequently, spatial navigation depends both on the type of cue available and on the type of learning provided. We investigated this interaction between spatial representation and memory by administering two different tasks (working memory, reference memory) using two different environmental cues (rectangular geometry, striped landmark) in mouse models of human genetic disorders: Prader-Willi syndrome (*PWScr^m+/p−^* mice, *n* = 12) and Beta-catenin mutation (Thr653Lys-substituted mice, *n* = 12). This exploratory study provides suggestive evidence that these models exhibit different abilities and impairments in navigating by boundary geometry and featural landmarks, depending on the type of memory task administered. We discuss these data in light of the specific deficits in cognitive and brain function in these human syndromes and their animal model counterparts.

## 1. Introduction

How do we encode and remember the vast amount of spatial information that we encounter as we navigate around a complex environment? Is it possible to tease apart the multitude of neurocognitive processes underlying spatial behavior? If so, could we identify specific impairments in neurological disorders that affect some of these processes more heavily than others? Decades of evidence from various fields of cognitive science converge on the finding that the brain organizes spatial information into at least two classes of environmental cues—boundary geometry and featural landmarks—that differentially influence navigation and spatial memory (see [1]). However, it is still unclear how these cues interact with learning processes to guide behavior.

Boundary-based spatial mapping has been well documented across all kinds of vertebrates, including fish, domestic chicks, rodents, and human toddlers. In contrast with the spontaneous and universal use of three-dimensional boundaries (e.g., walls or terrain structures), the influence of featural landmarks (e.g., object properties or two-dimensional surface markings) is more limited and variable across species and tasks (see [2,3], for review). First, while even young children spontaneously compute relative locations with respect to boundaries (e.g., “ten meters northwest of the distal wall”), they are more likely to use featural cues as direct markers of a location (e.g., “near the red object”) rather than as mapping cues. Second, although young children’s boundary-based navigation is invariant over both extrinsic and intrinsic factors such as the size of the environment or the age of the subject, their use of featural cues is more effective in larger spaces (e.g., [4]) and improves with age (e.g., [5]). Finally, feature use is much more susceptible to cuing, training, and individual experience (e.g., [6,7]). This is not only true in nonhuman animals but also in other species, in which the rearing conditions (i.e., the availability or absence of environmental cues in the home cage) do not affect their ability to map space solely in accordance with boundary geometry (fish: [8]; chicks: [9]; mice: [10]), and they can sometimes influence the relative control of featural cues on spatial behavior. 

The difference in spatial behavior according to boundary geometry and featural landmarks may be rooted in the distinctive cognitive and neural processing of the two types of cues. Evidence from human adults confirms findings from nonhuman animals and children and suggests that learning locations with respect to boundaries occurs “incidentally”, largely independently from past performance or reinforcement, while landmark-learning obeys the rules of reinforcement learning [11]. Dissociable neural correlates accompany these behavioral findings, with higher hippocampal activity associated with boundary-learning and higher striatal activity associated with landmark-learning [12]. The interpretation that boundary-coding relies on the hippocampus converges on decades of neurophysiological studies on the rodent brain. For many years, researchers have known that hippocampal place cells are highly sensitive to environmental boundaries [13,14,15], and the discovery of “boundary” or “border” cells in the subiculum and entorhinal cortex [16,17] has strengthened the hypothesis that representation of environmental boundaries in the hippocampal formation is critical for spatial mapping. 

If boundary geometry and features are separable in their neural representation and their dependence on reinforcement learning, it may be possible to identify specific impairments in human disorders involving hippocampal function or general learning processes. One example of this is in the case of Williams syndrome (WS), a genetic disorder caused by the deletion of a string of genes from chromosome 7 and characterized by a specific impairment in spatial abilities. Using a reorientation paradigm, researchers have shown that there is a clear distinction in the way landmarks and geometry are represented in WS [18]. A striking finding is that subjects with WS sometimes fail to encode environmental geometry altogether, while still being able to use a landmark to guide their navigation [19]. These results may be partially attributed to the fact that WS individuals have irregularities in their hippocampal morphology and function [20]. Genetically modified mouse models of this disorder have revealed that even partial deletions of the genes implicated in WS cause dysfunction in the hippocampus, down to the single-neuron level [21]. The combination of work on patient populations and animal models in WS provides a promising outlook for other neurological disorders associated with learning disabilities and hippocampal dysfunction.

The present study takes a step in this direction by exploring spatial learning and memory in two mouse models of human genetic disorders that are associated with impaired learning and hippocampal function: Prader-Willi syndrome (PW) and Beta-catenin gene mutation (which will be denoted hereafter as BC for Beta-catenin), respectively. PW is a neurodevelopmental disorder caused by a lack of paternally expressed imprinted genes in the PWS/AS (Prader-Willi Syndrome / Angelman Syndrome) locus of chromosome 15 in humans. The main symptoms are marked difficulties with attention, hypersomnolence, hyperactivity, overconsumption of food and repeated behaviors [22,23]. Individuals with Prader-Willi syndrome often have learning disabilities, difficulties in educational settings, and impaired reward-based learning [24,25]. On the other hand, a point mutation (Thr653Lys) in the Beta-catenin gene causes reduced hippocampal function, decreased interhemispheric connections, deficits in dendritic branching and long-term potentiation, along with morphological and sensorimotor irregularities. BC is associated with impaired performance in cognitive tasks [26], although it is not clear whether these effects are specific or general. Although rare, human patients identified with BC present similar profiles as mouse models with the same mutation. Mouse models of both PW and BC may be valuable for providing insight into the interaction between genes, the brain, and cognition. Specifically, by testing the two models on their use of boundaries or features in tasks that involve spontaneous or trained spatial memory, we may better understand the specific cognitive impairments caused by the neurological deficits (hippocampal or learning mechanisms) associated with the two disorders.

## 2. Present Task

**Figure 1:**
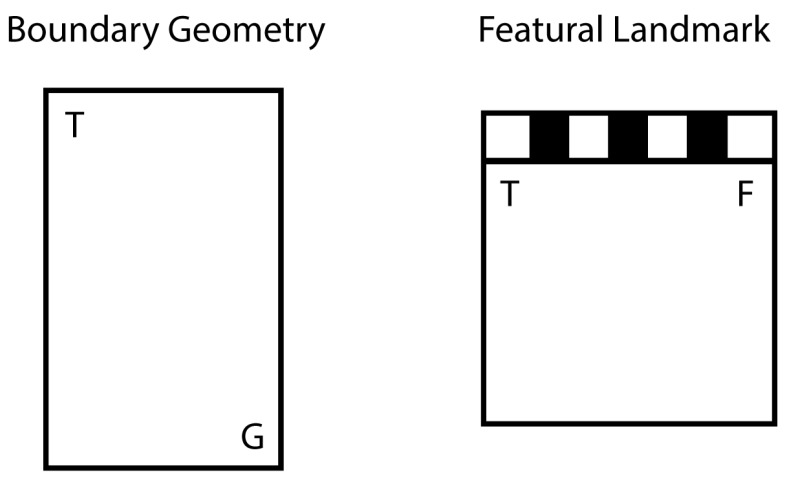
In the boundary geometry condition (left), the use of the rectangular arena geometry in the working-memory task is measured by comparing the total time spent at the target corner (T, which is inaccessible during the test) and its geometrically symmetrical corner (G) to the total time spent at the other two corners. In the reference-memory task, the time spent at G (before the animal finds the accessible T) is compared to the average time spent at the other two corners. As in the above example, if T is a corner with the “long wall on the left”, then G is the other corner with the same geometrical relation. In the featural landmark condition (right), the use of the striped wall in the working-memory task is measured by comparing the total time spent at the target corner (T) and the featurally symmetrical corner (F) to the total time spent at the other two corners. As in the above example, if T is a corner adjacent to the striped wall, then F is the other corner that is adjacent to the striped wall. In the reference-memory task, the time spent at F (before the animal finds the accessible T) is compared to the average time spent at the other two corners.

Here we tested mouse models of PW [27] and BC [26] in two tasks: working-memory navigation, with a varying goal location in each trial, and reference-memory navigation, with a consistently reinforced goal location across all trials. Each of these tasks was conducted across two different environments (see Figure 1): a uniformly gray rectangular arena (boundary geometry condition) and a square arena with three gray walls and one distinctive black/white striped wall (featural landmark condition).

Given that boundary-based navigation can be characterized as spontaneous and hippocampal, while feature-based navigation is associated with trained, reinforced learning, we might expect two different patterns of performance in the PW and BC mice. BC mice, with their hippocampal deficits, may be impaired in the working-memory task, especially in the boundary geometry condition, while unimpaired in learning to find a target in the reference-memory task, especially in the featural landmark condition. In contrast, if the PW mice have deficits in their reward-based learning mechanism, they may be impaired in the reference-memory task, especially with the featural landmark, while relatively unimpaired in the working-memory task, especially using boundary geometry.

## 3. General Methods and Materials

The behavioral task, first developed and published in [28], was an escape task using a testing arena partially filled with water. In a circular space formed by black curtains, a single central light source hung from the ceiling; above the light was a camera mounted directly onto the ceiling. The water-filled (5 mm deep) testing arena was placed on the floor at the center of the space. A uniformly gray-colored, rectangular arena (40 cm × 80 cm, height 20 cm) was used for testing boundary geometry. A square arena (40 cm × 40 cm, height 20 cm) with three gray walls and one striped (black/white, stripe thickness 4.5 cm) wall was used for testing feature-use.

Within the testing arena were four small boxes (one at each corner; 8 cm × 8 cm × 12 cm), one of which had a small opening that served as the target location. For each trial, the mouse was released into the arena and allowed to explore until it took shelter in the target location; the mouse was then transferred to a covered container, disoriented by slowly rotating the container, and then released back into the center of the arena, which was rotated 90 degrees to control for any asymmetries in the environment.

For the working-memory task, the target hole was varied across trials and blocked off (made inaccessible) for the test phase, when the mouse was returned to the arena immediately after disorientation. The search behavior of the mouse for 60 s following its release was recorded and analyzed. For the reference-memory task, the target remained open, accessible, and remained constant over trials. The search behavior of the mouse from its release to its arrival at the target corner was recorded and analyzed.

A corner choice was defined as any instance in which any part of the head/body of the mouse was within 3 cm from a corner box. Intercoder reliability was checked for 15% of the trials; the coders agreed 100% on the first approached corner and 90% on total number of seconds spent at each corner. For trials with initial coding discrepancies, an agreement was reached upon recoding.

All procedures were conducted under the Italian Policy, license issued to Valter Tucci on 19 June 2009, decreto N°106/2009-B at the Italian Institute of Technology.

## 4. Subjects

Subjects were male adult mice (eight to 12 weeks old). The first group of subjects was a mouse model of PWS used in this experiments presents with a deletion of MBII-85 in the ortholog chromosome 7 (*PWScr^m+/p−^*) (*n* = 12). The second group was the Beta-catenin (BC) mice with a point mutation (Thr653Lys) in the Beta-catenin gene (*n* = 12). A power analysis using the effect size and standard deviation measures from the geometry condition of the working memory task in Lee et al. (2015) (4.54 s difference in time spent at the correct vs. incorrect corners, standard deviation = 3.44), we would achieve a power of 0.98 with 12 subjects. A third group of subjects consisted of the littermate wild-type controls from both heterozygous groups (*n* = 19). Mice were housed in groups of 2–3 in standard mouse cages cleaned weekly and filled with fresh bedding and cardboard objects for enrichment). Mice were checked daily for their health and general well-being. All home cages were housed in the same room, which was maintained at 21–23 °C. The mice were provided standard food pellets (Mucedola 4RF21GLP, Certificate PF1610 for Mice and Rats, 13 mm × 25 mm) and water ad libitum, and put on a 12 h light/dark cycle. After completing this study, the mice were kept for use in other experiments.

Each subject was first tested in the working-memory task, with varied goal locations across trials, and then in the reference-memory task, which required learning a single rewarded location (see [28], for more details on the testing protocol). This was done in order to minimize interference of the single goal location learned in the reference-memory task. In each experiment, half of the subjects were tested in the geometry condition first, and the other six were tested in the feature condition first. A total of eight trials were administered for each of the working-memory tests of geometry and feature. A total of twenty trials were administered for each of the reference-memory tests of geometry and feature.

## 5. Results

**Figure 2:**
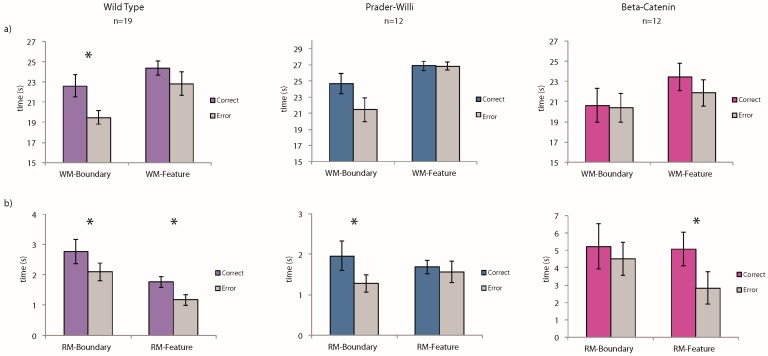
(**a**) Of the three groups, only the wild-type (WT) mice successfully used the boundary geometry to guide their navigation in the working-memory task (WM). Although the Prader-Willi (PW) group shows a clear trend in this direction, their preference for the geometrically correct corners was not significant. None of the groups showed a significant use of the striped landmark feature to guide their working-memory spatial behavior; (**b**) In the reference-memory task (RM) with one stable target location, the WT group relied on both the boundary geometry and landmark feature to find their goal. Consistent with their behavioral trend in the working-memory condition, PW mice successfully learned to use environmental geometry by the latter half of the 20 learning trials. However, this improvement did not apply to the feature condition. The Beta-catenin (BC) mice showed the opposite pattern from PW mice: BC mice learned to use the feature cue to guide navigation but continued to fail in the boundary condition. Asterisk denotes significant difference between correct choice and error (*p* < 0.05).

Working-memory task: We conducted planned comparisons of the average time spent at the correct vs. incorrect corners for each of the three strains: wild-type (WT), Prader-Willi (PW), Beta-catenin (BC). A “correct” response to geometry was defined as the total time spent at the two geometrically correct corners (the target corner and the rotationally equivalent corner, see Figure 1). A “correct” response to the landmark was defined as the total time spent at the two corners with the featural properties of the target corner (i.e., the two striped or the two unstriped corners) (see Figure 1). As shown in Figure 2a, only the WT group showed a significant preference for the geometrically correct corners in the working-memory task (WT: *t*(18) = 2.51, *p* = 0.02; PW: *t*(11) = 1.22, *p* = 0.25; BC: *t*(11) = 0.11, *p* = 0.91). Interestingly, none of the groups used the striped landmark to guide their spatial behavior in this working-memory task (WT: *t*(18) = 1.03, *p* = 0.32; PW: *t*(11) = 0.09, *p* = 0.93; BC: *t*(11) = 0.97, *p* = 0.35). A repeated-measures analysis of variance (ANOVA) with the three groups of mice and their performance did not reveal statistically significant interactions between the groups and their ability to use the spatial cues.

Reference-memory task: We conducted planned comparisons of the time spent at the correct vs. incorrect corners over the learning process for each of the three strains (WT, PW, BC). Because the reference-memory trials constantly left the target corner accessible, a “correct” response to geometry was defined as the total time spent at the rotationally equivalent corner before the animal found its way to the target corner. Similarly, a “correct” response to the landmark was defined as the total time spent at the corner with the same featural properties as the target corner before the animal found its way to the target corner (see Figure 1).

As shown in Figure 2b, the Prader-Willi mice successfully learned to use environmental geometry to find the one repeatedly reinforced goal. During the latter half of the 20 learning trials, they spent more time at the geometrically correct corner G than the average of time spent at the two geometrically incorrect corners (*t*(11) = 3.1, *p* = 0.01). In contrast, the BC mice failed to improve their performance across the 20 learning trials and did not show any preference for the geometrically correct corner, even during the latter half of the training (*t*(11) = 0.72, *p* = 0.49). The wild-type mice behaved consistently with their performance in the working-memory task and were guided by environmental geometry from the very start of the training (*t*(18) = 2.14, *p* = 0.04).

In the landmark condition, however, while the BC mice learned to use the striped wall to guide their navigation by the latter half of the learning trials, the Prader-Willi mice did not improve. The wild-type mice, as expected given the past findings [28], learned to use the featural cue (WT: *t*(18) = 3.19, *p* = 0.005; PW: *t*(11) = 0.62, *p* = 0.55; BC: *t*(11) = 2.24, *p* = 0.04). A repeated-measures ANOVA with the three groups of mice and their performance in both environmental conditions did not result in statistically significant interactions between the groups and the ability to remember the spatial cues.

## 6. Discussion

This exploratory study using mouse models of human genetic disorders has revealed an intriguing set of results that provides new insights into the relation between spatial representation and memory processes. We found that while PW mice were able to navigate using boundary geometry, they did not learn to use the featural landmark to guide them, at least over course of the training that we provided in the reference memory task. BC mice, on the other hand, were able to learn to navigate using the featural landmark, but did not improve in their navigation by boundary geometry over the trials. What might explain this double-dissociation?

The impairment in using boundary geometry in the BC mice may readily be attributed to their hippocampal dysfunctions at the neuronal level [26]. If their basic spatial navigation mechanisms which depend on environmental boundaries for mapping the environment are disrupted, we would predict that the BC mice are impaired in their use of the geometrical layout of boundaries to remember the target location. Their ability to learn the featural cues, on the other hand, would then be attributed to a relatively preserved neural circuit for reinforcement learning. In contrast, a generally normal hippocampal function, combined with learning deficits, would explain the opposite results (success with boundary mapping and failure with landmark learning) in PW syndrome. Nevertheless, it is crucial to note that the lack of interactions between mouse strain and performance makes these results preliminary and suggestive, rather than strongly conclusive.

By employing two memory tasks, we confirmed that WT mice, like other animals and human children (see [1]), remember locations with respect to boundary geometry in a reliable manner, not only in a reference-memory task but also in a working-memory task with a changing target location in every trial. Moreover, as in other animals, the mice learned to use a visual landmark to discriminate between locations near vs. far from the landmark, particularly in the reference-memory task, although they were not significantly above chance in the working-memory condition. This adds to the evidence that the use of featural landmarks is more variable, given that in a past study we found a partial sensitivity to the landmark in the working-memory task with even fewer animals (e.g., [28]). 

The PW and BC mice, despite showing trends in the working memory task, did not reach statistical significance in their performance. What may be the cause for the sole success of the WT group in the working-memory task? One might speculate that the ability to engage in spatial updating was compromised in the genetically modified mice. However, given that there were some clear trends in the working-memory task that were consistent with the reference-memory task, the lack of significant effects in the PW and BC groups (with 12 subjects) may be due to higher variability in their behavior in general. In other words, rather than reflecting spatial impairments specifically, perhaps working-memory performance is susceptible to perturbation from a range of general cognitive deficits, whether they are attentional, spatial, or memory-related.

## 7. Conclusions

Using two genetic mouse models of human neurological disorders, we have demonstrated that boundary geometry and featural landmarks may be dissociable in the ways they characterize and influence navigation behavior and spatial memory performance. The impaired boundary-based spatial working memory of the BC model and the impaired feature-based learning of the PW model reflect the specific corresponding neurological deficits associated with each of the disorders (hippocampal in BC and learning in PW). The distinctive behavioral phenotypes of PW and BC presented here open new doors for future research on identifying specific impairments in clinical populations and on the relation between genes, the brain, and behavior. More broadly, these behavioral profiles reflect the specificity and dissociability of neurocognitive processes that underlie spatial navigation and memory in vertebrates. 

## References

[1] Lee S.A., Spelke E.S. (2010). Two systems of spatial representation underlying navigation. Exp. Brain Res..

[2] Cheng K., Newcombe N.S. (2005). Is there a geometric module for spatial orientation? Squaring theory and evidence. Psychon. Bull. Rev..

[3] Tommasi L., Chiandetti C., Pecchia T., Sovrano V.A., Vallortigara G. (2012). From natural geometry to spatial cognition. Neurosci. Biobehav. Rev..

[4] Learmonth A.E., Newcombe N.S., Huttenlocher J. (2001). Toddlers’ use of metric information and landmarks to reorient. J. Exp. Child Psychol..

[5] Hermer-Vazquez L., Moffet A., Munkholm P. (2001). Langauge, space, and the development of cognitive flexibility in humans: The case of two spatial memory tasks. Cognition.

[6] Shusterman A., Lee S.A., Spelke E.S. (2011). Cognitive effects of language on human navigation. Cognition.

[7] Twyman A., Friedman A., Spetch M.L. (2007). Penetrating the geometric module: Catalyzing children’s use of landmarks. Dev. Psychol..

[8] Brown A.A., Spetch M.L., Hurd P.L. (2007). Growing in circles: Rearing environment alters spatial navigation in fish. Psychol. Sci..

[9] Chiandetti C., Vallortigara G. (2008). Is there an innate geometric module? Effects of experience with angular geometric cues on spatial re-orientation based on the shape of the environment. Anim. Cogn..

[10] Twyman A.D., Newcombe N.S., Gould T.J. (2013). Malleability in the development of spatial reorientation. Dev. Psychobiol..

[11] Doeller C.F., Burgess N. (2008). Distinct error-correcting and incidental learning of location relative to landmarks and boundaries. Proc. Natl. Acad. Sci. USA.

[12] Doeller C.F., King J.A., Burgess N. (2008). Parallel striatal and hippocampal systems for landmarks and boundaries in spatial memory. Proc. Natl. Acad. Sci. USA.

[13] Hartley T., Burgess N., Lever C., Cacucci F., O’Keefe J. (2000). Modeling place fields in terms of the cortical inputs to the hippocampus. Hippocampus.

[14] Hartley T., Lever C., Burgess N., O’Keefe J. (2014). Space in the brain: How the hippocampal formation supports spatial cognition. Philos. Trans. R. Soc. B: Biol. Sci..

[15] O’Keefe J., Burgess N. (1996). Geometric determinants of the place fields of hippocampal neurons. Nature.

[16] Lever C., Jeewajee A., Burton S., O’Keefe J., Burgess N. (2009). Hippocampal theta frequency, novelty, and behavior. Hippocampus.

[17] Solstad T., Boccara C.N., Kropff E., Moser M.B., Moser E.I. (2008). Representation of geometric borders in the entorhinal cortex. Science.

[18] Ferrara K., Landau B. (2015). Geometric and featural systems, separable and combined: Evidence from reorientation in people with Williams syndrome. Cognition.

[19] Lakusta L., Dessalegn B., Landau B. (2010). Impaired geometric reorientation caused by a genetic defect. Proc. Natl. Acad. Sci. USA.

[20] Meyer-Lindenberg A., Mervis C.B., Sarpal D., Koch P., Steele S., Kohn P., Marenco S., Morris C.A., Das S., Kippenhan S. (2005). Functional, structural, and metabolic abnormalities of the hippocampal formation in Williams syndrome. J. Clin. Investig..

[21] Osborne L.R. (2010). Animal models of Williams syndrome. Am. J. Med. Genet. Part C: Semin. Med. Genet..

[22] Cassidy S.B., Driscoll D.J. (2009). Prader-Willi Syndrome. Eur. J. Hum. Genet..

[23] Holm V.A., Cassidy S.B., Butler M.G., Hanchett J.M., Greenswag L.R., Whitman B.Y., Greenberg F. (1993). Prader-Willi syndrome: Consensus diagnostic criteria. Pediatrics.

[24] Gross-Tsur V., Landau Y.E., Benarroch F., Wertman-Elad R., Shalev R.S. (2001). Cognition, attention, and behavior in Prader-Willi syndrome. J. Child Neurol..

[25] Whittington J., Holland A., Webb T., Butler J., Clarke D., Boer H. (2004). Cognitive abilities and genotype in a population-based sample of people with Prader-Willi syndrome. J. Intell. Disabil. Res..

[26] Tucci V., Kleefstra T., Hardy A., Heise I., Maggi S., Willemsen M.H., Hilton H., Esapa C., Simon M., Buenavista M.T. (2014). Dominant β-catenin mutations cause intellectual disability with recognizable syndromic features. J. Clin. Investig..

[27] Lassi G., Maggi S., Balzani E., Cosentini I., Garcia-Garcia C., Tucci V. (2016). Working-for-food behaviors: A preclinical study in Prader-Willi mutant mice. Genetics.

[28] Lee S.A., Tucci V., Sovrano V.A., Vallortigara G. (2015). Working-memory and reference-memory tests of spatial navigation in mice (Mus musculus). J. Comp. Psychol..

